# Projected Impact of Vaccination Timing and Dose Availability on the Course of the 2014 West African Ebola Epidemic

**DOI:** 10.1371/currents.outbreaks.06e00d0546ad426fed83ff24a1d4c4cc

**Published:** 2014-11-21

**Authors:** David Fisman, Ashleigh Tuite

**Affiliations:** University of Toronto; Dalla Lana School of Public Health, University of Toronto, Toronto, Ontario, Canada

**Keywords:** ebola

## Abstract

Background: The 2014 West African Ebola outbreak has evolved into an epidemic of historical proportions and catastrophic scope. Prior outbreaks have been contained through the use of personal protective equipment, but such an approach has not been rapidly effective in the current epidemic. Several candidate vaccines have been developed against the Ebola virus, and are undergoing initial clinical trials. Methods: As removal of population-level susceptibility through vaccination could be a highly impactful control measure for this epidemic, we sought to estimate the number of vaccine doses and timing of vaccine administration required to reduce the epidemic size. Our base model was fit using the IDEA approach, a single equation model that has been successful to date in describing Ebola growth. We projected the future course of the Ebola epidemic using this model. Vaccination was assumed to reduce the effective reproductive number. We evaluated the potential impact of vaccination on epidemic trajectory under different assumptions around timing of vaccine availability. Results: Using effective reproductive (Re) number estimates derived from this model, we estimate that 3-4 million doses of vaccine, if available and administered, could reduce Re to 0.9 in the interval from January-March 2015. Later vaccination would be associated with a progressively diminishing impact on final epidemic size; in particular, vaccination to the same Re at or after the epidemic is projected to peak (April-May 2015) would have little impact on final epidemic size, though more intensive campaigns (e.g., Re reduced to 0.5) could still be effective if initiated by summer 2015. In summary, there is a closing window of opportunity for the use of vaccine as a tool for Ebola epidemic control. Conclusions: Effective vaccination, used before the epidemic peaks, would be projected to prevent tens of thousands of deaths; this does not minimize the ethical challenges that would be associated with wide-scale application of vaccines that have undergone only limited evaluation for safety and efficacy.

## Introduction

The 2014 West African Ebola epidemic has evolved into a human catastrophe of historical proportions 1 ; as of October 22, 2014, nearly 10,000 reported infections, and over 4,800 deaths, had been reported 13. While Ebola virus has a low reproductive number, and prior outbreaks have been controlled through use of personal protective equipment (PPE) and stringent burial practices 2 , the current epidemic has continued to grow despite these interventions. It has been noted that the speed with which Ebola treatment centres (ETC) can be constructed could easily be outstripped by growth in case numbers at rates that have been seen throughout the epidemic 11, and the most recent available World Health Organization situation report suggests that the number of staffed ETC beds in most-affected countries is remains smaller than promised, and is insufficient to permit care of all incident Ebola cases, with only 13% of cases in Sierra Leone cared for in ETC 19 .

An important driver of epidemic spread is the presence of abundant susceptible individuals who are both themselves at risk for infection, and who then infect others, resulting in the exponential growth in incidence that characterizes epidemics 3. While reducing transmission through PPE and case isolation can reduce the reproductive number to less than one (causing incidence to decelerate), an abundant supply of susceptible individuals means that such efforts need to be maintained indefinitely. By contrast, immunization in the context of an outbreak or epidemic has the attractive property of both preventing acquisition of infection by susceptible individuals, but also reducing the force of infection (rate at which susceptibles become infected, by reducing numbers of infectious cases); if sufficient fractions of the population are immunized, an epidemic should end rapidly.

Several promising candidate vaccines against Ebola virus are currently in clinical trials 12 . Although these trials are ongoing, and consequently, information about vaccine efficacy is unavailable, it would be desirable to approximate how many doses of vaccine would be required to substantially change the trajectory of this epidemic, and what the impact would be of delays in time to immunize substantial numbers of people. We used an existing mathematical model of the 2014 West African Ebola epidemic to model the quantity of a hypothetical highly effective vaccine needed to substantially decrease epidemic size, and also to simulate the impact of vaccination timing and dosing with a hypothetical vaccine on the future contours of the epidemic.

## Methods

The Effective Reproductive Number (R_e_) and Its Relation to Immunity

The basic reproductive number (R_0_) for a communicable disease can be defined as the average number of secondary infections produced by a primary infection in a wholly susceptible population, and in the absence of intervention. Many mathematical models heavily emphasize the role of susceptibility in maintaining transmission, such that the effective reproductive number (R_e_) is represented as:

*R_e_ = S·R_0_* [Eq. 1]

Here *S* is the susceptible fraction of the population. Using a well-fitting mathematical model (the Incidence Decay with Exponential Adjustment (IDEA) model 4 ^,^ 5 ), we observe that the West African Ebola epidemic is well-characterized as a process where R_e _is declining over time even as susceptibility in the population remains high; if *S* remains close to 1, declining *R_e_* must represent a combination of behavioral change and public health and medical intervention, or the presence of large numbers of unrecognized infections 6 . However, even in the presence of four-fold under-reporting (i.e., with 40,000 cases rather than approximately 10,000 cases in the region as of mid-October 2014), the fraction of immune individuals in the population would still be < 1%; as described below,* R_e_* has fallen by over 20%. In the presence of an *R_e_<R_0 _*when susceptibility is widespread, we can estimate the likely impact of a hypothetical 100% effective vaccine based on the relation:

*R_e_' = R_e_(1-V)* [Eq. 2]

*R_e_'* is the effective reproductive number in the presence of vaccination and V is the proportion of the population vaccinated. This relation can be rescaled based on vaccine efficacy (*E*) less than 100%, such that:

*R_e_' = R_e_(1-VE)* [Eq. 3]

IDEA Model and Projection of Re

We projected the future course of the epidemic using the IDEA model 4 ^,^ 5 , as described elsewhere. Briefly, this descriptive single equation model describes epidemics as processes characterized by exponential growth (a function of *R_0_*), with corresponding exponential "control" parameter (*d*), according to the relation:

*I_t_= [R_0_/(1+d)^t^]^t ^*[Eq. 4]

Here *t *is the epidemic "generation" (based on serial intervals, approximated as incubation + 1/2 the duration of infectiousness; we use 15 days for Ebola 7 ). *I_t_* is incident infections in a given generation, and *d* is a control parameter identified through fitting. Since the denominator is second order, incidence eventually approximates zero and the epidemic ends. As described elsewhere, our approximate end date for the 0^th^generation of the epidemic is January 6, 2014, and subsequent generation end dates are calculated at 15 day serial intervals from this date 7. Using this approach based on data available through August 22, 2014 we had previously estimated the global *R_0_* for the current Ebola epidemic to be approximately 1.7 5 , similar to estimates of other investigators 8 ^,^ 9 . We updated this model using World Health Organization case reports to October 18, 2014 (generation 19) obtained from a publicly accessible data repository maintained by Caitlin Rivers at the Virginia Tech [https://github.com/cmrivers/ebola]. We assumed the observations were Poisson distributed and used maximum likelihood methods to identify best-fit model parameters (using the *mle2* function in the *bbmle* R package 18 ), and modeled best- and worst-case scenarios based on upper- and lower-bound 95% confidence intervals (calculated using the *bbmle confint* function 18 ). Because *R_0_* and *d* estimates are positively correlated 5 (i.e., well-fitting models with higher *R_0_* have higher *d*) our worst case scenario was based on lower bound values for both parameters, while our best case scenario was based on upper bound parameter values for both parameters. Model fits were performed in the open source R statistical environment (http://www.r-project.org/).

We estimated time-dependent estimates of *R_e_* using model-generated projections of per-generation incidence. *R_e_* at some time *t* can be estimated as:

R_e_ = I_t_/(I_t-1_) [Eq. 5]

This is simply the ratio of incident case counts in succeeding generations. Based on time-dependent estimates of *R_e_* we calculated vaccine doses needed to drive *R_e'_* to 0.9 or to 0.5 in January, February, or March 2015 using Equation 2, and based on a population of 22 10 million persons (the approximate combined population of Guinea, Sierra Leone, and Liberia, where the epidemic has been centered). We assumed a 100% efficacious vaccine with a single dose required for immunity. For purposes of simplicity, we initially modeled the expected impact on epidemic scenarios when available doses could be given instantaneously. However, real-world vaccination programs would require time for implementation, and so we also explored more realistic scenarios with 10 million doses of vaccine administered in a rolling manner, at either 1 or 0.5 million doses per month, and starting in January, February, or March 2015. We also explored the impact of vaccination with vaccine with efficacy < 100%.

## Results

Model Fits , Projections and Estimated Re

Maximum likelihood estimates for *R_0_* and *d* were 1.79 (95% CI 1.78-1.81) and 0.00922 (95% CI 0.00879-0.00966) respectively, and the model fit well to available data (**Figure 1**).

**Model Fits to Observed Cumulative Ebola Case Counts:**
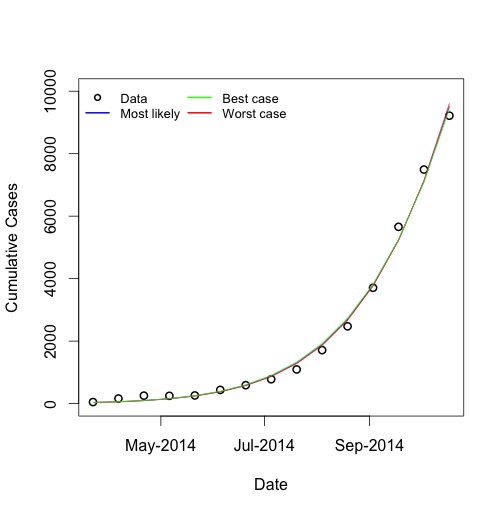
Model fits to cumulative Ebola virus case counts reported by World Health Organization, to October 18, 2014. Circles represent data, curves represent model fits for best-fit parameters, and for upper and lower bound parameters. Note that curves for most likely, best case, and worst case analyses display almost identical fits to data, but long term projections differ as below.

When most likely parameters were used to simulate the full course of the epidemic, we projected an epidemic that peaked in April or May 2015 for all scenarios, and ended in August 2016 (July 2016-September 2016 for best and worst case scenarios, respectively), with a final size of approximately 200,000 cases (160,000 - 260,000) (**Figure 2**).

**Model Projections of Most Likely, Best Case, and Worst Case Scenarios:**
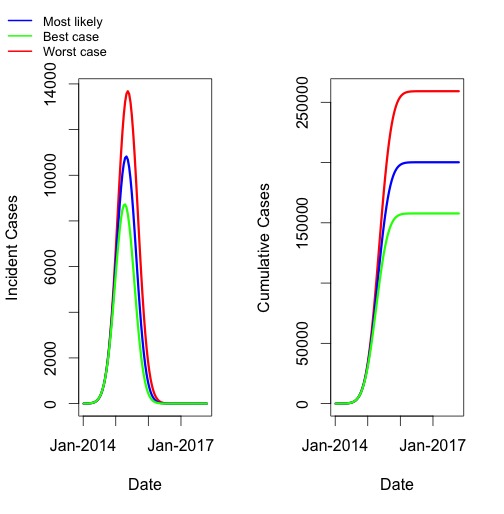
Long-term projections based on best-fit and upper and lower bound parameters. Blue curve represents most likely scenario, green curve represents best case, and red curve represents worst case. Incidence (per 15-day generation) is plotted on left; cumulative incidence on right.

Model derived estimates for R_e_ are presented in **Figure 3**. It can be seen that *R_e_* is estimated to fall steadily throughout the epidemic, approaching 1 when the epidemic peaks in April or May 2015. As the final size for the epidemic (in terms of recognized cases) is estimated to be approximately 1% of the regions' population, this magnitude of decline in *R_e _*could not be accounted for by accumulation of immune individuals, even in the presence of significant under-recognition and under-reporting of cases.

**Estimated Effective Reproductive Number for West African Ebola Outbreak by Date:**
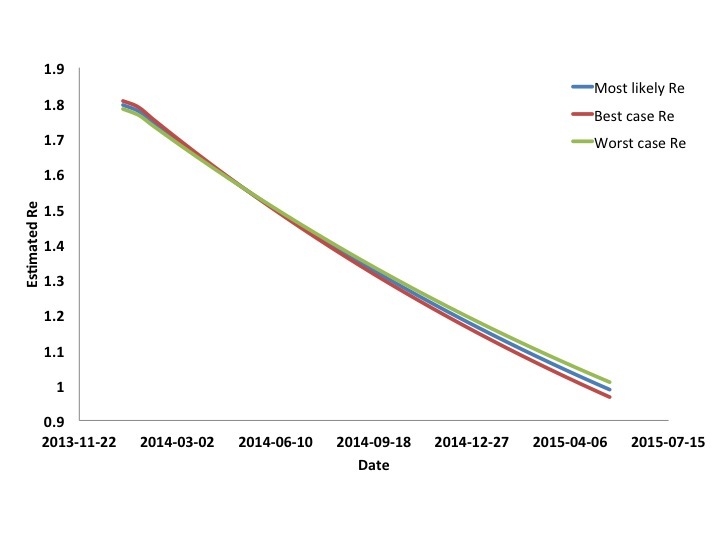
Curves are based on most likely, worst case, and best case scenarios. Initial reproductive numbers are equivalent to R0. Reproductive numbers approach 1 as the epidemic is projected to peak.

Based on Eq. 2 above, and assuming an at-risk population of 22 million individuals, we estimated the number of doses of vaccine that (if given instantaneously) would be necessary to reduce Re to 0.9, or to 0.5, by January, February or March 2015 in all three scenarios (**Figure 4)**.

**Estimated Dose Requirements for Reduction in Reproductive Number:**
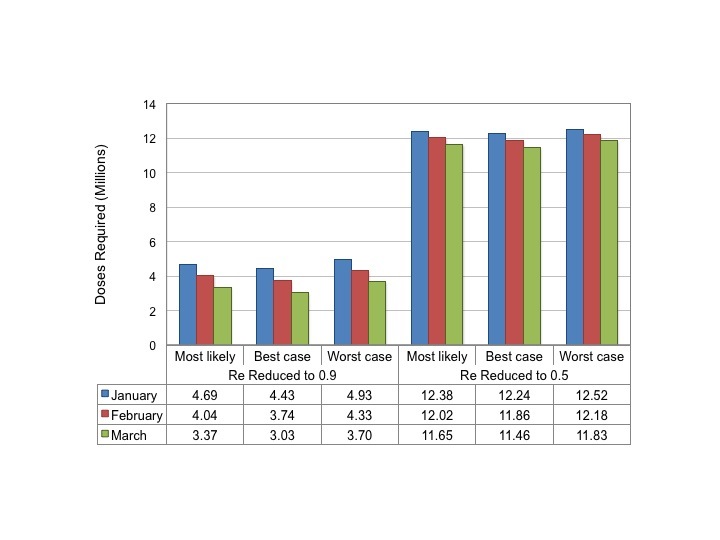
Dose requirements to reduce the reproductive number instantaneously to 0.9 (left side of panel) or 0.5 (right side of panel) are plotted based on month of administration. Numerical totals are presented in the table below graph. Note that for a vaccine with efficacy < 100% total doses can be estimated by dividing doses presented in the figure by vaccine efficacy.

Regardless of timing and scenario, between 3 and 4.9 million doses of a highly effective vaccine would be required to reduce *R_e_'* to 0.9; 11.5 to 12.5 million doses would be needed to reduce *R_e_'* to 0.5.

The projected impact of reduction of Re to 0.9 or 0.5 on epidemic contour for our most likely scenario is presented in **Figure 5**.

**Potential Impact of Vaccination to Reduce Reproductive Number:**
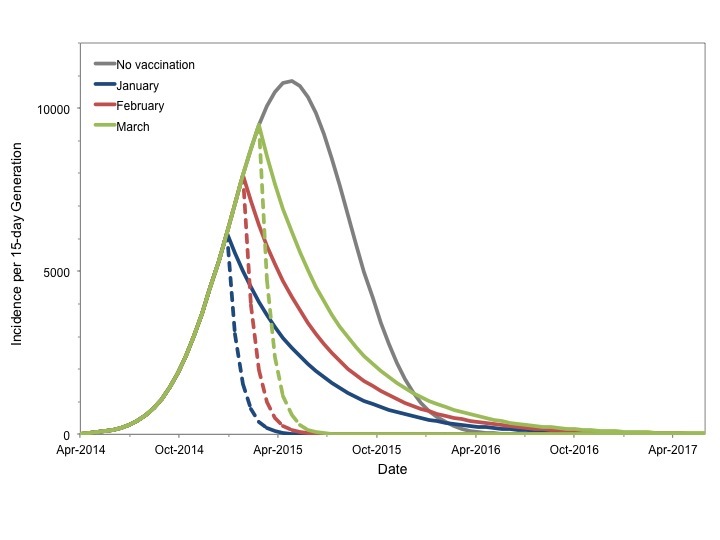
Epidemic curve projected for most likely scenario in the absence of vaccination is presented in grey. Hypothetical instantaneous vaccination sufficient to reduce Re to 0.9 (solid colored curves) or 0.5 (dashed curves) based on initiation in January (blue curve), February (red curves) or March 2015 (green curves) markedly changes projected course of the epidemic. Earlier and more intensive vaccination result in smaller final epidemic sizes.

Qualitatively similar results were seen for best and worst case scenarios (not shown). Vaccination markedly reduced projected epidemic size for all start dates. In all cases earlier vaccination resulted in a more substantial reduction in the final epidemic size than later vaccination. While vaccination sufficient to reduce *R_e_'* to 0.5 resulted in smaller epidemics than reducing *R_e_'* to 0.9 for a given start date, it is important to note that earlier immunization (January 2015) to reduce *R_e_*' to 0.9 resulted in a comparable final epidemic size to that achieved by reducing *R_e_*' to 0.5 in March 2015 (87,800 cases vs. 74,800 cases).

We performed additional analyses in which *R_e_' *was reduced to 0.9 or 0.5 as late as October 2015 (**Figure 6**). If vaccination reduces *R_e_'* to 0.9 and is initiated at or after the epidemic peak (April or May 2015), the reduction in total cases becomes negligible, as the epidemic's *R_e_* would be reduced below 1 even in the absence of vaccination. With more intensive vaccination to reduce *R_e_'*to 0.5, there is a wider time-window during which large reductions in epidemic size is possible, but again, after August 2015 even intensive vaccination results in little change in projected final epidemic size.

**Projected Impact of Reduction of Re' to 0.9 or 0.5 by Date:**
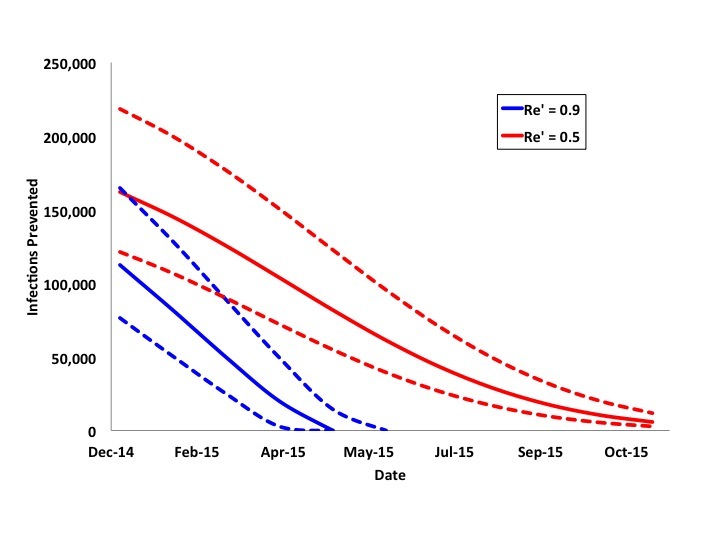
Curves represent infections prevented in most likely (solid curve) and best and worst case scenarios (dashed curves) with reduction of Re' to 0.9 (blue curves) or 0.5 (red curves) by vaccination on the date indicated on the x-axis. Epidemics are projected to peak in April or May 2015. More intensive vaccination prevents large numbers of cases even after the epidemic peaks, but ceases to be impactful if vaccination is not initiated before September 2015.

We performed an additional series of analyses based on our most likely scenario, in which 10 million doses of a hypothetical vaccine were available. Doses were administered either at a rate of 1 million doses per month over a 10-month period, or at a rate of 500,000 doses per month over a 20 month period. This "rolling vaccination" scenario could begin in January, February, or March of 2015 (**Figure 7**). Earlier and more intensive vaccination was most impactful.

**Projected Impact of Rolling Vaccination on Epidemic Size:**
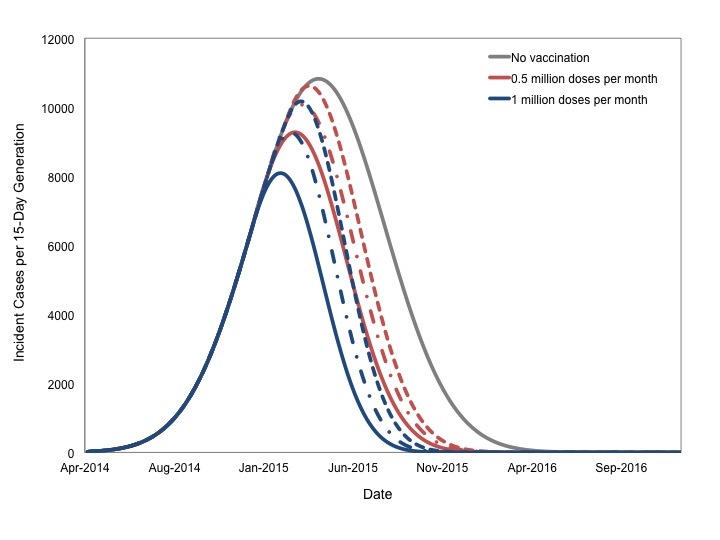
It is assumed that 10 million doses of vaccine are available for administration, and can be given either at a rate of 1 million doses monthly over 10 months (blue curves), or 500,000 doses monthly over 20 months (red curves), beginning in January (solid lines), February (dashed-dotted lines), or March (dashed lines) 2015.

As with instantaneous vaccination, earlier start dates resulted in markedly reduced final size; for a given start date more intensive vaccination resulted in more cases prevented (**Figure 8**).

**Cases Prevented Through Rolling Vaccination by Month of Initiation:**
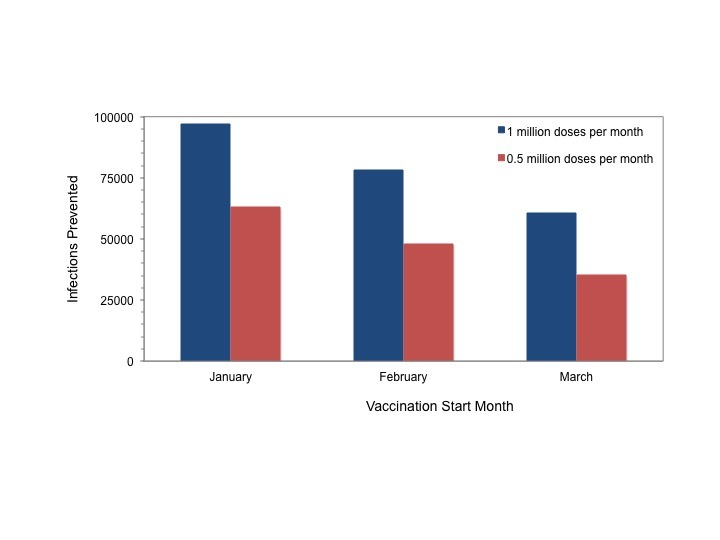
Total infections prevented by rolling vaccination with 1 million doses per month (blue bars) or 0.5 million doses per month, initiated in January, February or March 2015. Impact of vaccination declines with delay in initiation. More intensive vaccination is more effective for a given start date. Results are shown for the most likely scenario and were similar for the best and worst case scenarios.

We performed sensitivity analyses with varying vaccine efficacy (**Figure 9**). It can be seen that rolling vaccination strategies initiated as late as March 2015 could still impact the final epidemic size, even with vaccine efficacy substantially < 100%.

**Impact of Vaccine Efficacy on Projected Epidemic Trajectory:**
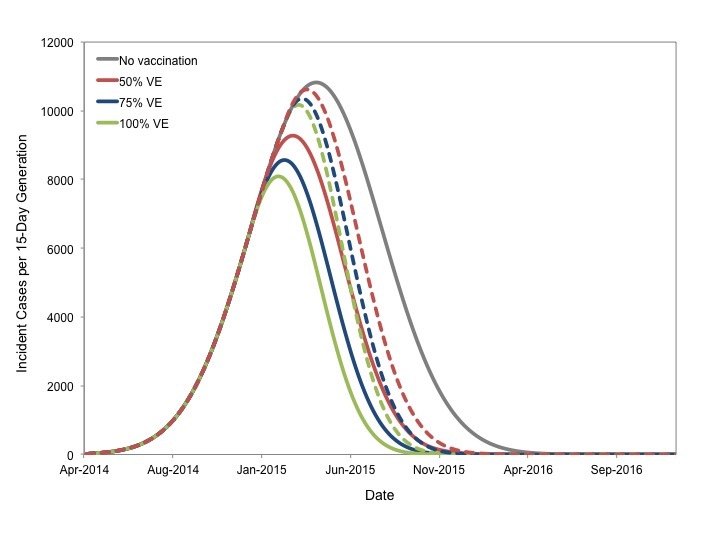
We assumed that 10 million doses of vaccine were administered at rate of 1 million doses per month, with varying vaccine efficacy (VE). Solid lines represent vaccination beginning in January 2015 and dashed lines represent vaccination initiated in March 2015.

## Discussion

While trials of vaccine for Ebola virus are ongoing, and consequently the estimates presented above are imprecise, our analysis offers four important qualitative results regarding vaccination as a tool for the control of the Ebola epidemic that may be helpful to manufacturers, governments, and health organizations:

1. We estimate that current non-vaccine interventions are reducing the reproductive number of Ebola in West Africa, even in the absence of large numbers of immune individuals. The final size of this epidemic is projected to be ~ 1% of the regional population. This means that reductions in reproductive number are unlikely to be due to large numbers of immune individuals, which in turn makes vaccination a highly attractive intervention to help control this epidemic.
2. While our estimates are by necessity imprecise, we can project that the availability of > 3 million highly efficacious vaccine doses by January 2015 could substantially reduce final size if these could be provided to individuals in a timely way. We do not minimize the challenges this will pose to vaccine distribution, not least of which may be cold chain in a resource-poor part of the world. However, we also note that the ability to immunize care providers themselves could facilitate a large scale increase in the availability of both local and international volunteers and staff.
3. The timing of vaccine administration is key to an impactful vaccine program. To date our projections of the course of this epidemic have been quite accurate. If they continue to be, the epidemic will peak in the spring of 2015, most likely in April or May. Effective vaccination sufficient to reduce the reproductive number to < 1 prior to the epidemic peak would prevent tens of thousands of infections, and given the high case fatality of Ebola virus disease, tens of thousands of death.
4. Vaccination must begin as soon as possible if it is to be impactful. In this analysis, the impact of delaying initiation of vaccination by even a month or two substantially decreased the potential of this intervention to save lives. Indeed, the impact of a less intense (*R_e_'* = 0.9) vaccination program beginning in January was nearly equivalent to a far more intense (*R_e_'* = 0.5) vaccination program delayed until March. The reason for this relates to the number of prevalent cases at the time of initiation. By March, the epidemic is near to peaking, and since *R_e_'* represents the ratio of current to future cases, a larger number of current cases will result in substantial incident case numbers even in the generation following immunization. Simply put, the diminished impact of late vaccination relates to the higher force-of-infection (risk of infection among susceptibles) in place at the time the vaccine is introduced 17. If vaccination is delayed until *R_e_* declines to < 1 without vaccination, the epidemic will subside with or without vaccination, and the window of opportunity for substantial reduction in epidemic size using vaccination will have closed.

Like any mathematical model, the model we use is subject to limitations, including data inputs of uncertain quality (e.g., World Health Organization case counts), its descriptive rather than explicitly mechanistic nature, and limited information on the efficacy of candidate vaccines, including the number of doses that can be given per manufactured vial. We do not wish to imply that manufacture of sufficient doses of vaccine is the only barrier to the implementation of a successful vaccine program; other hurdles related to implementation and cold chain are likely to exist; furthermore we note that even in the event that a vaccine is created, other challenges (including accurate and timely case identification, provision of safe and effective medical care, and adequate protection of caregivers and those involved in burial) are likely to remain. Furthermore, apparent successes have been seen in regional control of the epidemic with non-pharmaceutical interventions; notably, ETC beds in heavily populated areas of Liberia have been empty in October 2014, perhaps as a result of implementation of safer burial practices and other interventions 20.

Nonetheless, the epidemic continues to grow in West Africa as a whole, and the qualitative projections presented here are important, as they suggest that the window for effective use of vaccination will close rapidly if vaccine is not deployed. This in turn raises important ethical questions, as a vaccine program deployed in early 2015 would of necessity lack the usual level of certainty about vaccine efficacy and safety 16 . We would argue that the catastrophic nature of the Ebola epidemic may make widespread use of novel and little tested vaccines, with close monitoring, and ideally in the context of stepped-wedge clinical trials 15 , may in fact be the only ethical alternative if sufficient vaccine is available.

**Addendum:** During the time period while this paper has been under review, an additional generation of case counts has become available for incorporation into our analysis (case counts as of November 2, 2014). As incorporation of these data into our model did not change our projections we have presented the analysis based on October 18, 2014 case counts; we anticipate updating our model regularly and will post important changes in projected epidemic contour as comments on this paper.

## References

[1] Farrar JJ, Piot P. The Ebola emergency--immediate action, ongoing strategy. N Engl J Med. 2014 Oct 16;371(16):1545-6. PubMed PMID:25244185. 25244185 10.1056/NEJMe1411471

[2] Kerstiëns B, Matthys F. Interventions to control virus transmission during an outbreak of Ebola hemorrhagic fever: experience from Kikwit, Democratic Republic of the Congo, 1995. J Infect Dis. 1999 Feb;179 Suppl 1:S263-7. PubMed PMID:9988193. 9988193 10.1086/514320

[3] Fisman D. Modelling an influenza pandemic: A guide for the perplexed. CMAJ. 2009 Aug 4;181(3-4):171-3. PubMed PMID:19620267. 19620267 10.1503/cmaj.090885 PMC2717691

[4] Fisman DN, Hauck TS, Tuite AR, Greer AL. An IDEA for short term outbreak projection: nearcasting using the basic reproduction number. PLoS One. 2013;8(12):e83622. PubMed PMID:24391797. 24391797 10.1371/journal.pone.0083622 PMC3877403

[5] Fisman D, Khoo E, Tuite A. Early Epidemic Dynamics of the West African 2014 Ebola Outbreak: Estimates Derived with a Simple Two-Parameter Model. PLOS Currents Outbreaks. 2014 Sep 8. Edition 1. doi: 10.1371/currents.outbreaks.89c0d3783f36958d96ebbae97348d571 PMC4169344 25642358

[6] Ebola control: effect of asymptomatic infection and acquired immunity Steve E Bellan,Juliet R C Pulliam,Jonathan Dushoff,Lauren Ancel Meyers The Lancet - 16 October 2014 DOI: 10.1016/S0140-6736(14)61839-0 10.1016/S0140-6736(14)61839-0 PMC4829342 25390569

[7] Ebola virus disease in West Africa--the first 9 months of the epidemic and forward projections. N Engl J Med. 2014 Oct 16;371(16):1481-95. PubMed PMID:25244186. 25244186 10.1056/NEJMoa1411100 PMC4235004

[8] Nishiura H, Chowell G. Early transmission dynamics of Ebola virus disease (EVD), West Africa, March to August 2014. Euro Surveill. 2014 Sep 11;19(36). PubMed PMID:25232919. 25232919 10.2807/1560-7917.es2014.19.36.20894

[9] Althaus CL. Estimating the Reproduction Number of Ebola Virus (EBOV) During the 2014 Outbreak in West Africa. PLOS Currents Outbreaks. 2014 Sep 2. Edition 1. doi: 10.1371/currents.outbreaks.91afb5e0f279e7f29e7056095255b288. 10.1371/currents.outbreaks.91afb5e0f279e7f29e7056095255b288 PMC4169395 25642364

[10] The World Bank. Data, 2014. Available via the Internet at data.worldbank.org/country. Last accessed October 22, 2014.

[11] Joseph A Lewnard, Martial L Ndeffo Mbah, Jorge A Alfaro-Murillo, Frederick L Altice, Luke Bawo, Tolbert G Nyenswah, Alison P Galvani. Dynamics and control of Ebola virus transmission in Montserrado, Liberia: a mathematical modelling analysis. The Lancet Infectious Diseases 24 October 2014(Article in Press DOI: 10.1016/S1473-3099(14)70995-8) 10.1016/S1473-3099(14)70995-8 PMC4316822 25455986

[12] Cohen J, Kupferschmidt K. Leaked documents reveal behind-the-scenes Ebola vaccine issues. Science Insider, October 23, 2014. Available via the Internet at http://news.sciencemag.org/health/2014/10/leaked-documents-reveal-behind-scenes-ebola-vaccine-issues. Last accessed October 27, 2014.

[13] World Health Organization. Ebola Response Roadmap Situation Report, October 22, 2014. Available via the Internet at http://apps.who.int/iris/bitstream/10665/137091/1/roadmapsitrep22Oct2014_eng.pdf?ua=1. Last accessed October 27, 2014.

[14] Greenhalgh D, Dietz K. Some bounds on estimates for reproductive ratios derived from the age-specific force of infection. Math Biosci. 1994 Nov;124(1):9-57. PubMed PMID:7827426. 7827426 10.1016/0025-5564(94)90023-x

[15] Scott JM, deCamp A, Juraska M, Fay MP, Gilbert PB. Finite-sample corrected generalized estimating equation of population average treatment effects in stepped wedge cluster randomized trials. Stat Methods Med Res. 2014 Sep 29. PubMed PMID:25267551. 25267551 10.1177/0962280214552092 PMC4411204

[16] World Health Organization. Ethical considerations for use of unregistered interventions for Ebola virus disease (EVD): Summary of the panel discussion. August 12, 2014. Available via the Internet at http://www.who.int/mediacentre/news/statements/2014/ebola-ethical-review-summary/en/. Last accessed October 27, 2014.

[17] Vynnycky E, White RG (2010) An introduction to infectious disease modelling. Oxford ; New York: Oxford University Press, 370 pp.

[18] Bolker B, R Development Core Team (2011). bbmle: Tools for general maximum likelihood estimation. R package version 1.0.17

[19] World Health Organization. Ebola response roadmap - Situation report. November 19, 2014. Available via the Internet at http://www.who.int/csr/disease/ebola/situation-reports/en/. Last accessed November 21, 2014.

[20] Nyenswah TG, Westercamp M, Kamali AA, Qin J, Zielinski-Gutierrez E, Amegashie F, Fallah M, Gergonne B, Nugba-Ballah R, Singh G, Aberle-Grasse JM, Havers F, Montgomery JM, Bawo L, Wang S, Rosenberg R. Evidence for Declining Numbers of Ebola Cases — Montserrado County, Liberia, June–October 2014. Morb Mort Weekly Rep: November 14, 2014 / 63(Early Release);1-5. Available via the Internet at www.cdc.gov/mmwr/preview/mmwrhtml/mm63e1114a2.htm. Last accessed November 21, 2014.

